# Early recovery patterns differ between medially congruent and posterior‐stabilized total knee arthroplasty, with comparable 2‐year outcomes: A triple‐blinded randomized clinical trial

**DOI:** 10.1002/jeo2.70861

**Published:** 2026-07-30

**Authors:** Fardis Vosoughi, Arash Sharafat Vaziri, Farzad Khashami, Zahra Vahdati, Iman Menbari Oskouie, Hossein Nematian

**Affiliations:** ^1^ Department of Orthopaedics and Trauma Surgery, Shariati Hospital Tehran University of Medical Sciences Tehran Iran; ^2^ Sports Orthopaedics and Arthroscopy Research (SOAR) Center, Tehran University of Medical Sciences Tehran Iran; ^3^ Center for Orthopedic Trans‐Disciplinary Applied Research Tehran University of Medical Sciences Tehran Iran

**Keywords:** medial congruent, medial pivot, posterior‐stabilized, recovery trajectory, total knee arthroplasty

## Abstract

**Purpose:**

Whether medially congruent (MC) total knee arthroplasty (TKA) provides clinically meaningful advantages over posterior‐stabilized (PS) designs remains controversial. In this randomized trial, early recovery profiles and 2‐year outcomes were compared between Persona® MC and PS TKA.

**Methods:**

In this triple‐blinded randomized controlled trial, 82 patients undergoing primary TKA for Kellgren–Lawrence Grade IV osteoarthritis were allocated to receive a Persona® MC or PS system. The primary endpoint was Knee injury and Osteoarthritis Outcome Score–Activities of Daily Living (KOOS‐ADL). Secondary outcomes included range of motion, visual analogue scale (VAS) pain, Forgotten Joint Score‐12 (FJS‐12), satisfaction and complications. Follow‐up was 2 years. Group, time and group × time interaction were assessed using repeated‐measures models.

**Results:**

Both groups improved over time. Significant group × time interactions were observed for ROM, VAS pain, KOOS‐ADL and FJS‐12, indicating different recovery profiles. MC knees had greater flexion at 2 weeks (95.3° vs. 83.9°, *p* < 0.001), and flexion contracture at 2 weeks was observed only in the PS group (0/41 vs. 4/41). Conversely, PS patients reported lower pain scores at 2 weeks (4.75 vs. 6.17, *p* < 0.001) and 6 weeks (1.92 vs. 2.97, *p* < 0.001). Later flexion favoured PS, including at 2 years (118.8° vs. 113.9°, *p* < 0.001). MC patients reported statistically higher KOOS‐ADL and FJS‐12 scores at 1 and 2 years; however, between‐group differences did not exceed established minimum clinically important difference thresholds. Satisfaction was high in both groups and complications were infrequent.

**Conclusion:**

Persona® MC and PS TKA showed different recovery profiles rather than clear superiority of either design. MC‐TKA was associated with better early flexion and fewer observed early flexion contractures, whereas PS TKA was associated with lower early postoperative pain and greater flexion at later follow‐up. MC showed statistically higher KOOS‐ADL and FJS‐12 scores, but these differences remained below minimum clinically important difference thresholds. Both designs provided substantial 2‐year improvement without clinically meaningful between‐group differences in patient‐reported outcomes.

**Level of Evidence:**

Level I.

## Graphical abstract

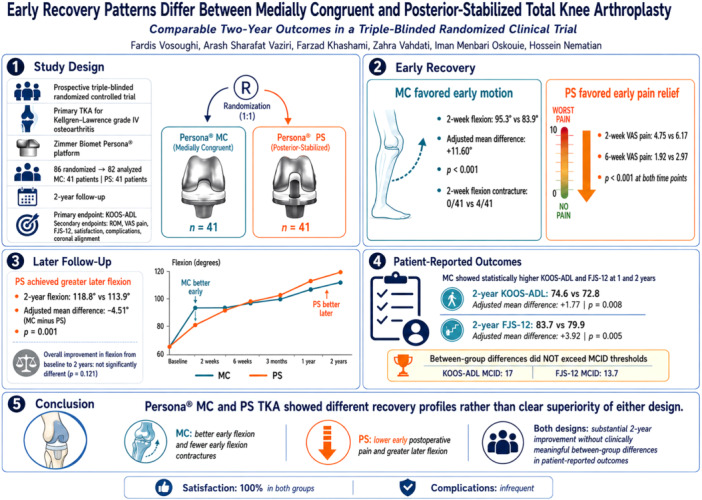

**Abbreviations**
FJS‐12: Forgotten Joint Score‐12
KOOS‐ADL: Knee injury and Osteoarthritis Outcome Score–Activities of Daily Living
MC: medially congruent
MCID: minimum clinically important difference
PCA: patient‐controlled analgesia
PCL: posterior cruciate ligament
PS: posterior‐stabilized
ROM: range of motion
SNOSE: sequentially numbered, opaque, sealed envelopes
TKA: total knee arthroplasty
VAS: visual analogue scale

## INTRODUCTION

Total knee arthroplasty (TKA) provides reliable pain relief and functional improvement for patients with advanced knee osteoarthritis; however, residual symptoms, dissatisfaction and persistent joint awareness remain clinically relevant concerns after otherwise successful surgery [5, 19, 23]. Implant design may contribute to these outcomes by influencing knee stability, range of motion (ROM), pain and the patient's perception of the replaced joint [15].

Medially congruent (MC) inserts were developed to enhance medial compartment stability through a more conforming medial articulation while allowing relatively greater lateral compartment mobility [1, 3, 4, 6, 18]. In contrast, posterior‐stabilized (PS) designs provide stability through a post‐cam mechanism after posterior cruciate ligament (PCL) substitution. Although these concepts are biomechanically distinct, the clinical relevance of these differences remains uncertain.

Previous comparative studies and systematic reviews have reported mixed results when MC or medial‐stabilized designs were compared with PS or other conventional TKA designs [2, 9, 16, 17, 22, 27, 32]. Some studies have suggested modest advantages in patient‐reported outcomes, joint awareness or early function, whereas others have found no clinically meaningful differences in ROM or functional scores. Moreover, although several studies have included early postoperative assessments, the specific temporal pattern of recovery after MC versus PS TKA within the same implant platform and under standardized perioperative conditions remains insufficiently characterized.

This distinction is clinically important because recovery after TKA is not defined only by the final 2‐year outcome. Early flexion, flexion contracture and postoperative pain may influence rehabilitation participation and patient‐perceived recovery, whereas later patient‐reported outcomes may better reflect function and joint awareness after recovery has plateaued. Therefore, evaluating both early recovery profile and 2‐year outcomes may provide a more complete understanding of whether implant design affects the course of postoperative recovery or only the final magnitude of improvement.

The aim of this triple‐blinded randomized controlled trial was to compare early clinical recovery and 2‐year patient‐reported outcomes between Persona® MC and Persona® PS TKA. Specifically, it was assessed whether implant design influenced early knee ROM and flexion contracture, early postoperative pain, 2‐year function measured by the Knee injury and Osteoarthritis Outcome Score Activities of Daily Living subscale (KOOS‐ADL) and joint awareness measured by the Forgotten Joint Score‐12 (FJS‐12). It was hypothesized that MC and PS TKA would demonstrate different early recovery profiles, but no clinically meaningful difference in 2‐year patient‐reported outcomes.

## MATERIALS AND METHODS

### Study design and reporting

This study was structured as a prospective, triple‐blind, randomized controlled trial with parallel group allocation and a 1:1 allocation ratio. Medially congruent total knee arthroplasty (MC‐TKA) was compared with posterior‐stabilized total knee arthroplasty (PS‐TKA) using the same implant platform. The study was reported in accordance with the CONSORT guidelines for randomized trials, and the completed CONSORT checklist is provided as supplementary material [10]. Bias was minimized through concealed allocation, blinded outcome assessment, blinded statistical analysis, standardized perioperative care and predefined outcome assessment time points.

### Participants

Between September 2022 and August 2023, consecutive candidates for primary TKA due to Kellgren–Lawrence Grade IV primary knee osteoarthritis were screened at a tertiary referral centre. Inclusion criteria were age 50–80 years, American Society of Anesthesiologists physical Status I–III and body mass index (BMI) between 18 and 35 kg/m^2^. Patients with BMI > 35 kg/m^2^ were excluded to reduce heterogeneity related to perioperative risk, rehabilitation capacity, wound‐related complications and implant loading, all of which could independently influence early recovery and patient‐reported outcomes. Exclusion criteria included prior ipsilateral knee surgery or infection, inflammatory arthritis (e.g., rheumatoid arthritis), neurologic disease affecting mobility, severe deformity requiring custom implants or inability or unwillingness to complete the planned 2‐year follow‐up.

### Randomization and blinding

Participants were randomly assigned to receive either MC‐TKA (experimental group) or PS‐TKA (comparator group). The allocation sequence was generated using computer‐generated blocked randomization with randomly varying block sizes and a 1:1 allocation ratio (www.randomization.com). Allocation was concealed using sequentially numbered, opaque, sealed envelopes (SNOSE) until participants were enroled and assigned to interventions (allocation concealment). The allocation process was managed by an independent study nurse who was not involved in recruitment, surgery, follow‐up assessment or statistical analysis. Eligible patients who provided consent were enroled before allocation was revealed.

Blinding was maintained for patients, outcome assessors and the statistician throughout the study period. The operating surgeon could not be blinded due to the nature of the intervention. To reduce potential surgeon‐related performance bias, all procedures were performed by the same experienced arthroplasty surgeon using a standardized surgical technique and perioperative protocol, and the surgeon was not involved in postoperative outcome assessment or statistical analysis.

### Surgical protocol

All TKAs were performed by a single experienced arthroplasty surgeon using a standard anterior midline incision and medial parapatellar arthrotomy. Patients received the Zimmer Biomet Persona® MC or Persona® PS system (Zimmer Biomet) according to randomization. Patellar resurfacing was not performed in either group. Patelloplasty was performed in all patients using a standardized technique, including removal of patellar osteophytes and marginal reshaping when required. Circumpatellar denervation was performed routinely in all patients.

Bone cuts and soft‐tissue balancing were performed using a hybrid technique, with gap balancing in extension and measured resection in flexion. Posterior referencing was used to preserve posterior femoral offset. The PCL was routinely resected in both groups. Tibial slope was set according to manufacturer recommendations (5° for MC and 3° for PS).

Perioperative management was standardized: low‐dose spinal anaesthesia, preoperative cefazolin (2 g) and tranexamic acid (1 g), tourniquet use, local infiltration analgesia and no drain placement. Postoperative care included early ambulation with a walker, a multimodal analgesic regimen intended to minimize opioids (including celecoxib and short‐course oxycodone with patient‐controlled analgesia (PCA) during hospitalization) and thromboprophylaxis with rivaroxaban 10 mg daily for 30 days.

### Outcome assessment

Demographics, clinical history and baseline knee function were recorded preoperatively. The primary outcome was the KOOS‐ADL subscale (0–100), selected a priori and used for sample size calculation. The Persian version of the KOOS has previously been culturally adapted and validated in Iranian patients, and the ADL subscale was therefore used as the primary patient‐reported functional outcome [24]. Secondary outcomes included ROM, visual analogue scale (VAS) pain score, FJS‐12, patient satisfaction, complications and coronal alignment.

Follow‐up assessments were performed at 2 weeks, 6 weeks, 3 months, 1 year and 2 years. ROM and VAS were recorded at all follow‐up visits. Pain intensity was assessed using a standard 0–10 VAS, with Persian verbal anchors explained to each participant by the blinded assessor. KOOS‐ADL was assessed preoperatively, at 6 weeks, 1 year and 2 years, whereas FJS‐12 was assessed at 1 and 2 years. The Persian version of the FJS‐12 has previously demonstrated acceptable validity and reliability in Iranian patients undergoing arthroplasty and other knee‐related conditions [20, 21].

At the 2‐year follow‐up, patient satisfaction was assessed using a five‐category global satisfaction question with the following choices: ‘very dissatisfied’, ‘dissatisfied’, ‘no opinion’, ‘satisfied’ and ‘very satisfied’. This item was used as a global descriptive measure of satisfaction and was not intended to represent a validated multi‐item satisfaction instrument. Overall satisfaction with surgery was evaluated using this method, and results were compared between groups.

All assessments were conducted by a trained research assistant blinded to group allocation. Knee ROM was measured as active flexion and extension using a standard long‐arm goniometer. Measurements were performed with the patient in the supine and seated positions using standardized anatomical landmarks and were recorded to the nearest 5°. All ROM measurements were performed by the same blinded assessor. Standardized protocols were followed for measurement, and data were independently verified by the study supervisor [20, 21, 24].

### Radiographic assessment

Preoperative coronal deformity and postoperative coronal alignment were assessed using standing full‐length hip‐to‐ankle radiographs. Varus alignment was measured as the mechanical hip–knee–ankle angle using standardized digital measurement software. Measurements were recorded to the nearest 1°. Postoperative coronal alignment was measured using the same method at the final follow‐up. Radiographic measurements were performed by one blinded assessor.

### Definition of recovery profile

The term ‘early recovery profile’ was operationalized as the temporal pattern of change in ROM, flexion contracture and pain during the early postoperative period, assessed using repeated‐measures models and group × time interaction effects. This term was not intended to imply a formal time‐to‐event, slope or area‐under‐the‐curve analysis. When significant group × time interactions were identified, between‐group comparisons at specific follow‐up time points were used to describe the direction and timing of differences between implant designs.

### Sample size

Sample size estimation was based on KOOS‐ADL as the primary outcome. Previous randomized studies comparing implant designs in TKA have reported standard deviations of approximately 12–18 points for KOOS‐derived outcomes. Assuming a conservative standard deviation of 15 points and aiming to detect a moderate effect size of 0.55, corresponding to an approximate between‐group difference of 8 points, with 80% power and a two‐sided alpha of 0.05, the minimum required sample size was 36 patients per group. This calculation was intended to detect statistically significant between‐group differences and was not powered specifically to detect the established minimum clinically important difference. To compensate for potential attrition, the target sample size was increased to at least 40 patients per group, and 43 patients were randomized to each group.

### Statistical analysis

Statistical analysis was performed using IBM SPSS Statistics version 25.0 (IBM Corp.). Continuous variables are presented as mean ± standard deviation, and categorical variables are presented as frequency and percentage. Baseline continuous variables were compared using independent‐samples *t* tests or Mann–Whitney *U* tests, depending on distribution. Categorical variables were compared using chi‐square or Fisher's exact tests, as appropriate.

To account for the longitudinal nature of the data and within‐subject correlations across repeated measurements, clinical and patient‐reported outcomes were analysed using repeated‐measures models. For each continuous outcome, including ROM, VAS, KOOS‐ADL and FJS‐12, time was included as the within‐subject factor and implant design (MC vs. PS) as the between‐subject factor. The primary effects of interest were the main effect of group, the main effect of time and the group × time interaction. The group × time interaction was used to determine whether the temporal pattern of recovery differed between implant designs.

The original analysis was performed as a repeated‐measures framework in SPSS rather than as a likelihood‐based linear mixed‐effects model with a separately selected covariance matrix. Therefore, no additional covariance structure, such as first‐order autoregressive or unstructured covariance, was specified. Within‐subject correlation was accounted for through the repeated‐measures design. This approach was selected to evaluate temporal outcome patterns while reducing reliance on multiple isolated point‐by‐point comparisons.

Because baseline varus alignment differed between groups, adjusted sensitivity analyses were performed by including baseline varus angle and the baseline value of each respective outcome as covariates in the mixed‐model repeated‐measures analyses. This adjustment was undertaken to account for potential confounding effects and to improve internal validity.

Patients lost to 2‐year follow‐up were not included in the final analysed cohort. Missing values were not imputed. Analyses were performed using available data from the analysed cohort. When significant group × time interactions were identified, post hoc pairwise comparisons with Bonferroni adjustment were performed to explore between‐group differences at individual follow‐up time points.

Established minimum clinically important difference (MCID) thresholds were used to interpret the clinical relevance of between‐group differences: 17 points for KOOS‐ADL [14] and 13.7 points for FJS‐12 [7]. All tests were two‐sided, and *p* values of <0.05 were considered statistically significant.

### Ethical aspects

The study was approved by the Institutional Review Board of Tehran University of Medical Sciences (IR.TUMS.SHARIATI.REC.1402.070) and was registered before patient enrolment in the Iranian Registry of Clinical Trials (IRCT20191222045857N2). All participants provided written informed consent before enrolment. The study was conducted in accordance with the approved protocol and relevant ethical guidelines.

## RESULTS

### Baseline characteristics

**Figure 1:**
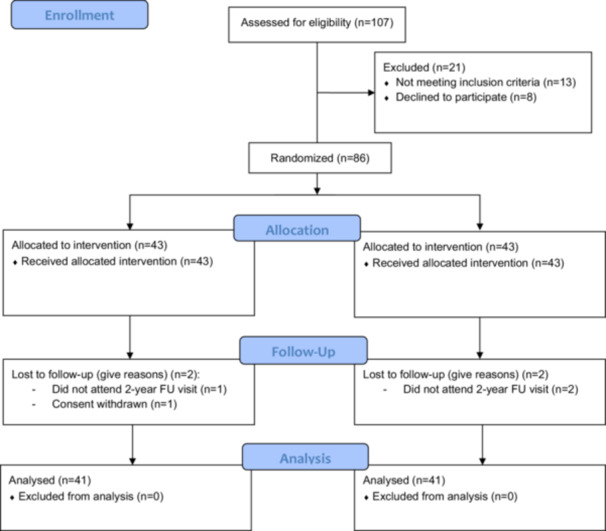
CONSORT diagram of patient enrolment, recruitment and follow‐up. FU, follow‐up.

Of 107 patients assessed for eligibility, 21 were excluded (13 did not meet eligibility criteria and 8 declined participation) and 86 were randomized. Two patients in each group were lost to follow‐up, leaving 82 patients available for the final analysis: 41 in the MC group and 41 in the PS group. No patient who completed follow‐up was excluded from the analysis (Figure 1).

Baseline demographic and clinical characteristics were generally comparable between groups, including age, sex, BMI, baseline ROM, KOOS‐ADL score and VAS pain score. However, preoperative varus deformity was significantly greater in the MC group than in the PS group (15.4 ± 2.8° vs. 12.2 ± 4.4°; mean difference, 3.2°; 95% CI, 1.6–4.9; *p* < 0.001). This baseline imbalance was therefore accounted for in adjusted sensitivity analyses (Table 1). Furthermore, operating time was also compared between groups, and no statistically significant difference was observed.

**Table 1:** Baseline demographics and preoperative status.

Variable	MC TKA (*n* = 41)	PS TKA (*n* = 41)	Difference, MC–PS (95% CI)	SMD	*p* value
Age, mean ± SD (years)	68.9 ± 8.0	66.8 ± 6.5	2.1 (−1.1 to 5.3)	0.28	0.067
Female sex, *n* (%)	36 (87.8%)	39 (95.1%)	−7.3% (−19.3 to 4.7)	−0.26	0.216
BMI, mean ± SD (kg/m^2^)	28.9 ± 2.3	29.6 ± 4.1	−0.7 (−2.1 to 0.8)	−0.20	0.353
Recurvatum, *n* (%)	2 (4.9%)	1 (2.4%)	2.4% (−5.7 to 10.5)	0.13	0.662
Flexion contracture, *n* (%)	25 (61.0%)	29 (70.7%)	−9.8% (−30.2 to 10.7)	−0.21	0.130
Flexion, mean ± SD (°)	103.3 ± 9.3	105.1 ± 8.7	−1.8 (−5.8 to 2.1)	−0.20	0.249
VAS pain score, mean ± SD	8.4 ± 0.7	8.6 ± 0.7	−0.3 (−0.6 to 0.0)	−0.37	0.086
KOOS‐ADL score, mean ± SD	16.6 ± 2.5	16.6 ± 3.0	0.1 (−1.2 to 1.3)	0.02	0.937
Varus angle, mean ± SD (°)	15.4 ± 2.8	12.2 ± 4.4	3.2 (1.6–4.8)	0.86	0.001
Operation time, mean ± SD (min)	74.5 ± 10.9	76.9 ± 7.9	−2.4 (−6.6 to 1.8)	−0.25	0.062

*Note*: Values are presented as mean ± standard deviation or *n* (%). Differences are presented as mean differences for continuous variables and risk differences for categorical variables. Positive values indicate higher values in the MC group.

Abbreviations: BMI, body mass index; KOOS‐ADL, Knee injury and Osteoarthritis Outcome Score Activities of Daily Living; MC, medially congruent; PS, posterior‐stabilized; ROM, range of motion; SD, standard deviation; SMD, standardized mean difference; TKA, total knee arthroplasty; VAS, Visual Analogue Scale.

### Radiographic coronal alignment

Postoperative coronal alignment improved substantially in both groups. At final follow‐up, residual varus alignment was similar between the MC and PS groups (2.4 ± 1.7° vs. 2.5 ± 1.6°; mean difference, −0.1°; 95% CI, −0.8 to 0.6; *p* = 0.790). After adjustment for baseline varus alignment, the between‐group difference in postoperative varus alignment remained non‐significant (adjusted mean difference, −0.3°; 95% CI, −1.1 to 0.5; *p* = 0.418).

### ROM and flexion contracture

A significant main effect of time was demonstrated by repeated‐measures analysis of knee ROM (*p* < 0.001), indicating substantial postoperative improvement in both groups over the follow‐up period. A significant group × time interaction was also observed, reflecting different recovery trajectories between the MC and PS implants.

Knee flexion changed significantly over time in both groups, with different temporal patterns between implant designs. Greater early flexion was observed in the MC group at 2 weeks compared with the PS group (95.3 ± 8.4° vs. 83.9 ± 5.9°; mean difference, 11.4°; 95% CI, 8.2–14.6; *p* < 0.001). This difference remained significant after adjustment for baseline flexion and baseline varus alignment (adjusted mean difference, 11.60°; 95% CI, 8.10–15.09; *p* < 0.001).

By 6 weeks, flexion was similar between groups (100.7 ± 7.2° vs. 101.2 ± 6.0°; *p* = 0.740), and the adjusted between‐group difference was negligible (adjusted mean difference, 0.00°; 95% CI, −3.16 to 3.17; *p* = 0.999). At later time points, greater flexion was achieved in the PS group than in the MC group. At 3 months, flexion was 108.0 ± 6.8° in the MC group and 114.4 ± 5.5° in the PS group, with an adjusted mean difference of −5.64° for MC minus PS (95% CI, −8.62 to −2.67; p < 0.001). At 2 years, flexion was 113.9 ± 6.5° in the MC group and 118.8 ± 4.0° in the PS group, and the adjusted difference remained in favour of PS‐TKA (adjusted mean difference, −4.51°; 95% CI, −7.09 to −1.92; *p* = 0.001).

**Figure 2:**
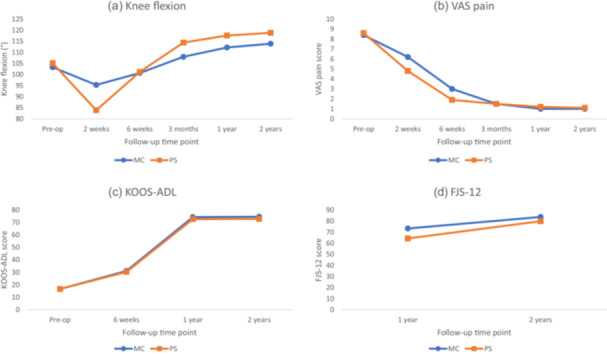
Chronological trends in knee flexion (a), VAS pain score (b), KOOS‐ADL (c) and FJS‐12 (d) scores in the MC and PS groups over 2 years of follow‐up. Panels show mean values at each assessment time point. KOOS‐ADL was assessed preoperatively, at 6 weeks, 1 year and 2 years. FJS‐12 was assessed at 1 and 2 years only. FJS‐12, Forgotten Joint Score‐12; KOOS‐ADL, Knee injury and Osteoarthritis Outcome Score–Activities of Daily Living; MC, medially congruent; PS, posterior‐stabilized; VAS, Visual Analogue Scale.

Despite these differences in recovery patterns, the overall magnitude of improvement in knee flexion from baseline to final follow‐up did not differ significantly between groups (PS: preoperative = 105.12°, postoperative = 118.82°, mean change = 13.7°; MC: preoperative = 103.29°, postoperative = 113.90°, mean change = 10.61°; *p* = 0.121), suggesting comparable 2‐year functional gains (Table 2; Figure 2).

**Table 2:** Functional and patient‐reported outcomes.

Outcome	Time point	MC TKA	PS TKA	Unadjusted MD, MC–PS (95% CI)	Adjusted model estimate, MC–PS (95% CI)	Adjusted *p* value
ROM flexion (°)	Pre‐op	103.3 ± 9.3	105.1 ± 8.7	−1.8 (−5.8 to 2.1)	—	—
	2 weeks	95.3 ± 8.4	83.9 ± 5.9	11.4 (8.2–14.6)	11.60 (8.10–15.09)	<0.001
	6 weeks	100.7 ± 7.2	101.2 ± 6.0	−0.5 (−3.4 to 2.4)	0.00 (−3.16 to 3.17)	0.999
	12 weeks	108.1 ± 6.8	114.4 ± 5.5	−6.3 (−9.1 to −3.6)	−5.64 (−8.62 to −2.67)	<0.001
	1 year	112.2 ± 6.1	117.6 ± 4.3	−5.4 (−7.7 to −3.0)	−4.34 (−6.86 to −1.82)	0.001
	2 years	113.9 ± 6.5	118.8 ± 4.0	−4.9 (−7.3 to −2.5)	−4.51 (−7.09 to −1.92)	0.001
VAS pain score	Pre‐op	8.4 ± 0.7	8.6 ± 0.7	−0.3 (−0.6 to 0.0)	—	—
	2 weeks	6.2 ± 1.4	4.8 ± 1.3	1.4 (0.8–2.0)	1.42 (0.81–2.03)	<0.001
	6 weeks	3.0 ± 0.9	1.9 ± 0.8	1.1 (0.7–1.4)	0.99 (0.59–1.40)	<0.001
	12 weeks	1.5 ± 0.6	1.5 ± 0.6	0.0 (−0.2 to 0.3)	0.01 (−0.31 to 0.33)	0.966
	1 year	1.0 ± 0.4	1.2 ± 0.4	−0.2 (−0.4 to 0.0)	−0.18 (−0.38 to 0.01)	0.069
	2 years	1.0 ± 0.4	1.1 ± 0.4	−0.1 (−0.3 to 0.1)	−0.19 (−0.39 to 0.01)	0.064
KOOS‐ADL score	Pre‐op	16.6 ± 2.5	16.6 ± 3.0	0.1 (−1.2 to 1.3)	—	—
	6 weeks	31.1 ± 4.4	30.3 ± 4.5	0.9 (−1.1 to 2.8)	1.26 (−0.89 to 3.40)	0.247
	1 year	74.3 ± 2.9	72.7 ± 2.4	1.6 (0.4–2.8)	1.46 (0.19–2.74)	0.025
	2 years	74.6 ± 2.9	72.8 ± 2.5	1.8 (0.6–3.0)	1.77 (0.47–3.06)	0.008
FJS‐12 score	1 year	73.3 ± 5.8	64.3 ± 5.7	9.1 (6.5–11.6)	10.17 (7.46–12.87)	<0.001
	2 years	83.7 ± 6.0	79.9 ± 5.8	3.8 (1.2–6.4)	3.92 (1.21–6.62)	0.005

*Note*: Values are presented as mean ± standard deviation unless otherwise indicated. Mean differences are calculated as MC minus PS; therefore, positive values favour MC and negative values favour PS. Adjusted model estimates were adjusted for baseline varus alignment and the baseline value of the corresponding outcome when available. FJS‐12 estimates were adjusted for baseline varus alignment because baseline FJS‐12 was not available.

Abbreviations: CI, confidence interval; FJS‐12, Forgotten Joint Score‐12; KOOS‐ADL, Knee injury and Osteoarthritis Outcome Score Activities of Daily Living; MC, medially congruent; MD, mean difference; PS, posterior‐stabilized; ROM, range of motion; TKA, total knee arthroplasty; VAS, visual analogue scale.

Flexion contracture at 2 weeks was observed in no patients in the MC group and in four patients in the PS group (0/41 vs. 4/41, 9.8%). No flexion contracture was observed in either group from 6 weeks onward (Figure 2).

### Pain outcomes

VAS pain scores showed a significant main effect of time (*p* < 0.001), indicating a marked reduction in pain following surgery in both groups. In contrast to the early flexion findings, lower early postoperative pain scores were reported in the PS group. At 2 weeks, VAS pain was 6.17 ± 1.37 in the MC group and 4.76 ± 1.30 in the PS group (mean difference, 1.41; 95% CI, 0.83–2.00; *p* < 0.001). This difference remained significant after adjustment for baseline VAS and baseline varus alignment (adjusted mean difference, 1.42; 95% CI, 0.81–2.03; *p* < 0.001).

At 6 weeks, VAS pain remained higher in the MC group than in the PS group (2.98 ± 0.91 vs. 1.93 ± 0.75; mean difference, 1.05; 95% CI, 0.68–1.42; *p* < 0.001), with a similar adjusted estimate (adjusted mean difference, 0.99; 95% CI, 0.59–1.40; *p* < 0.001). From 3 months onward, adjusted between‐group differences in VAS pain were no longer statistically significant (Table 2; Figure 2).

### Patient‐reported outcomes

A significant main effect of time was demonstrated for KOOS‐ADL scores (*p* < 0.001), reflecting significant functional improvement in both implant groups throughout the study period. The group × time interaction was significant, reflecting different patterns of functional recovery. At 6 weeks, there was no significant between‐group difference after adjustment for baseline KOOS‐ADL and baseline varus alignment (adjusted mean difference, 1.26; 95% CI, −0.89 to 3.40; *p* = 0.247). At 1 and 2 years, KOOS‐ADL scores were statistically higher in the MC group. At 2 years, KOOS‐ADL was 74.6 ± 2.9 in the MC group and 72.8 ± 2.5 in the PS group. The adjusted mean difference at 2 years was 1.77 points in favour of MC‐TKA (95% CI, 0.47–3.06; *p* = 0.008). However, this difference was well below the established MCID threshold of 17 points [14] (Table 2).

A significant main effect of time was revealed by FJS‐12 analysis, with progressive improvement in joint awareness in both groups. A significant group × time interaction was observed, favouring the MC implant. FJS‐12 scores were higher in the MC group at both follow‐up time points. At 1 year, FJS‐12 was 73.3 ± 5.8 in the MC group and 64.3 ± 5.7 in the PS group. After adjustment for baseline varus alignment, the mean difference remained significant (adjusted mean difference, 10.17; 95% CI, 7.46–12.87; *p* < 0.001). At 2 years, FJS‐12 was 83.7 ± 6.0 in the MC group and 79.9 ± 5.8 in the PS group, with an adjusted mean difference of 3.92 points (95% CI, 1.21–6.62; *p* = 0.005). Both the 1‐year and 2‐year between‐group differences were below the established MCID threshold of 13.7 points [7] (Table 2).

### Satisfaction

At the 2‐year follow‐up, all analysed patients in both groups were either satisfied or very satisfied with the procedure. The satisfaction rate was therefore 100% in both groups.

### Complications

Complications were infrequent. Postoperative knee pain with crepitus developed in one patient in the PS group (1/41, 2.4%), whereas no such event occurred in the MC group (0/41, 0%). No periprosthetic joint infection, instability, cardiopulmonary complication or periprosthetic fracture was observed in either group.

## DISCUSSION

The principal finding was that MC and PS TKA demonstrated different early recovery profiles rather than clear clinical superiority of either implant design. MC‐TKA was associated with greater knee flexion at 2 weeks and fewer observed early flexion contractures, whereas PS‐TKA was associated with lower pain scores during the first 6 postoperative weeks and greater flexion at later follow‐up intervals. Although KOOS‐ADL and FJS‐12 scores were statistically higher in the MC group at 1 and 2 years, the between‐group differences did not exceed established MCID thresholds. Therefore, the main clinical interpretation is that both designs provided substantial 2‐year improvement, with implant design influencing selected aspects of the recovery pattern rather than producing a clinically meaningful overall advantage.

The finding of greater early flexion in the MC group is consistent with the theoretical rationale of MC designs. MC inserts are intended to enhance medial compartment stability while permitting relatively greater lateral compartment mobility, which may facilitate confidence during early motion and rehabilitation [1, 3, 4, 6, 18, 23]. However, this early flexion advantage did not persist throughout follow‐up. By 6 weeks, flexion was similar between groups, and at 3 months, 1 year and 2 years, greater flexion was achieved in the PS group. This finding argues against an overly simple interpretation that MC‐TKA provides globally faster recovery. Rather, the data suggest that implant design may affect different dimensions of recovery in different directions: MC favoured early flexion, while PS favoured early pain relief and later flexion.

The lower early pain scores in the PS group are clinically relevant and should be considered alongside the early ROM findings. Although the mechanism for this observation cannot be determined from the present study, it demonstrates that early recovery is multidimensional and cannot be inferred from ROM alone. Pain in the first weeks after TKA may influence patient perception, rehabilitation tolerance and satisfaction with early recovery. This is consistent with recent evidence emphasizing the importance of multimodal pain management and anaesthetic strategies, including low‐dose intravenous ketamine protocols, in improving postoperative pain control after TKA [30, 31]. Therefore, the results do not support a one‐sided conclusion favouring MC‐TKA during early recovery; instead, they indicate divergent recovery profiles, with MC and PS designs each showing advantages in different early postoperative domains.

Previous systematic reviews and meta‐analyses comparing medial pivot, medial‐stabilized or MC designs with PS or conventional designs have reported inconsistent findings [11, 16, 27, 32]. Some studies have suggested modest functional or complication‐related advantages for medial‐stabilized concepts, whereas others have shown no clinically meaningful differences in ROM or patient‐reported outcome measures (PROMs). The present findings are broadly consistent with this mixed literature. The present study supports the view that final outcomes after modern TKA are generally favourable with both designs, while also suggesting that serial early follow‐up can reveal time‐dependent differences that may be missed when only final endpoints are compared.

Additional evidence is provided by the present trial regarding the limited literature specifically comparing Persona MC and Persona PS inserts. Previous Persona‐specific studies were retrospective and therefore more vulnerable to selection bias, surgeon‐related heterogeneity and incomplete control of perioperative variables [8, 13]. Broadly similar 2‐year outcomes across polyethylene conformity groups were reported by Indelli et al. (with only marginal flexion differences) but limited by its retrospective design and lacked serial early follow‐up that would illuminate recovery kinetics [13]. Satisfactory early outcomes with MC bearings were reported by Frye et al., but the nonrandomized design, uneven distribution across multiple bearing types, multi‐surgeon variability and limited follow‐up constrained inference [8]. By using randomized allocation, blinded outcome assessment, a single implant platform and standardized perioperative care, the present study provides stronger evidence that observed differences reflect recovery patterns associated with insert design rather than major differences in surgical pathway or implant system.

Comparison with the 2022 systematic reviews by Xu et al. and Shi et al. also provides context [27, 32]. No consistent difference in postoperative ROM between MC and PS designs was reported in both reviews, which is broadly consistent with the present 2‐year observation of comparable overall ROM improvement. However, better early flexion and less flexion contracture were observed at the first follow‐up in the MC group, followed by later time points in which greater flexion was observed in the PS group at selected visits. Importantly, despite these time‐specific differences, the overall improvement from baseline did not differ meaningfully between groups, supporting the conclusion that long‐term ROM outcomes are broadly comparable.

PROM findings across prior studies have also been mixed. Xu et al. reported no consistent differences in Knee Society Score (KSS), Oxford Knee Score (OKS), Western Ontario and McMaster Universities Arthritis Index (WOMAC), FJS or Kujala scores, whereas Shi et al. found better WOMAC outcomes with MC designs [27, 32]. The PROM findings require careful interpretation. KOOS‐ADL scores were statistically higher in the MC group at 1 and 2 years, and FJS‐12 scores also favoured MC at both time points. However, the magnitude of these differences remained below established MCID thresholds for both KOOS‐ADL and FJS‐12 [7, 14]. This distinction is important. Statistical significance may reflect low variability and adequate study power, but it does not necessarily indicate a difference that patients would perceive as clinically meaningful. Therefore, the PROM results should be interpreted as showing a small statistical advantage in favour of MC‐TKA, particularly for joint awareness, rather than definitive clinical superiority.

The higher FJS‐12 scores observed in the MC group may suggest reduced awareness of the replaced knee during daily activities. The FJS‐12 has been proposed as a useful high‐ceiling measure after arthroplasty because it may discriminate among well‐functioning knees when broader functional scores approach saturation [28, 29]. Recent evidence has also used FJS‐12 as an outcome measure to evaluate joint awareness after TKA in relation to implant positioning and rotational mismatch [33]. Prior comparative studies have also suggested that medial‐stabilized or medially conforming designs may be associated with improved joint awareness or patient‐reported function in selected cohorts [12, 25]. Nevertheless, because the between‐group FJS‐12 differences in the present study remained below the MCID threshold, these findings should be viewed as subtle differences in joint perception rather than evidence of a major patient‐perceived advantage.

The 100% satisfaction rate in both groups should be interpreted with caution. Although all analysed patients reported being either satisfied or very satisfied at 2 years, this finding is higher than typically reported after contemporary TKA (often ~90%–95%) [5, 23]. Several factors may have contributed, including strict selection criteria, exclusion of patients lost to follow‐up, high baseline disability, cultural and expectation‐related factors and the use of a simple five‐category global satisfaction question. This satisfaction question was not a dedicated validated multi‐item satisfaction instrument, and ceiling effects may have limited its ability to detect more subtle between‐group differences. High satisfaction may therefore reflect both substantial postoperative improvement and contextual expectation effects rather than a definitive statement about comparative implant performance. Therefore, satisfaction results should be considered supportive of generally favourable outcomes in both groups, but not as definitive evidence that dissatisfaction was absent in the broader target population.

Regarding complications, a lower incidence of patellar clunk/crepitus with MC designs was suggested by Xu et al., and lower overall/local complication rates in MC knees were reported by Shi et al., without differences in prosthesis‐related or systemic complications [27, 32]. In the present cohort, complications were infrequent in both groups. One patient in the PS group developed postoperative knee pain with crepitus, and no periprosthetic joint infection, instability, cardiopulmonary complication or periprosthetic fracture occurred in either group. Because the study was not powered to detect uncommon complications, particularly patellofemoral complications such as crepitus or clunk, no firm conclusions can be drawn regarding differences in complication risk between implant designs. The observed complication profile should therefore be interpreted descriptively.

This study has several limitations. First, although randomization was used, baseline varus deformity was significantly greater in the MC group. Adjusted sensitivity analyses accounting for baseline varus alignment did not materially change the main findings, but residual confounding cannot be completely excluded. Second, the two inserts were implanted according to manufacturer recommendations, including different tibial slope targets: 5° for MC and 3° for PS. Because tibial slope can influence knee flexion, gap balance and postoperative kinematics, some ROM differences may reflect implant‐specific surgical philosophy rather than insert conformity alone [26]. Third, the operating surgeon could not be blinded. Although all procedures were performed by the same experienced surgeon using a standardized protocol and the surgeon was not involved in postoperative assessment or statistical analysis, surgeon‐related performance bias cannot be entirely excluded. Fourth, the single‐surgeon and single‐centre design improves procedural consistency but may limit generalizability to other surgeons, centres, alignment strategies and rehabilitation pathways. Fifth, patients with BMI > 35 kg/m^2^ were excluded to reduce heterogeneity and perioperative risk; therefore, the findings may not be generalizable to patients with severe obesity, who represent an important subgroup in primary TKA. Sixth, the sample size was adequate for detecting moderate differences in the primary PROM but was not powered for uncommon complications or subgroup analyses. Finally, the 2‐year follow‐up provides important early clinical outcome data but should not be interpreted as midterm or long‐term implant performance.

Despite these limitations, the study has notable strengths. These include its randomized design, allocation concealment, blinded outcome assessment, blinded statistical analysis, equal group sizes, use of the same implant platform, standardized perioperative care and serial assessment of both early clinical recovery and 2‐year patient‐reported outcomes. Taken together, the findings suggest that Persona MC and PS TKA both provide substantial clinical improvement at 2 years. Their differences are best understood as domain‐specific and time‐dependent: MC showed better early flexion and slightly higher later PROMs, whereas PS showed lower early pain and greater later flexion. These findings may be useful for patient counselling regarding expected recovery patterns, but they do not support a conclusion that either design is clinically superior overall.

## CONCLUSIONS

In this triple‐blinded randomized trial comparing Persona MC and PS TKA, substantial 2‐year clinical improvement was achieved with both designs. MC‐TKA was associated with greater early flexion and fewer observed early flexion contractures, whereas PS‐TKA was associated with lower early postoperative pain and greater flexion at later follow‐up. Although KOOS‐ADL and FJS‐12 scores were statistically higher in the MC group at 1 and 2 years, these differences did not exceed established MCID thresholds. These findings suggest different recovery profiles rather than clinically meaningful overall superiority of either implant design.

## AUTHOR CONTRIBUTIONS

All authors who participated in this study made substantial contributions to the conception or design of the work, acquisition, analysis and interpretation of data. They drafted the work or revised it critically for important intellectual content; approved the version to be published and agree to be accountable for all aspects of the work in ensuring that questions related to the accuracy or integrity of any part of the work are appropriately investigated and resolved. Material preparation, data collection and analysis were performed by Fardis Vosoughi, Arash Sharafat Vaziri, Farzad Khashami, Zahra Vahdati, Iman Menbari Oskouie and Hossein Nematian. The first draft of the manuscript was written by Hossein Nematian and all authors commented on previous versions of the manuscript. All authors read and approved the final manuscript.

## FUNDING INFORMATION

The authors have no funding to report.

## CONFLICT OF INTEREST STATEMENT

The authors declare no conflicts of interest.

## ETHICS STATEMENT

Institutional review board approval was obtained before the start of the study (Registration Code: IRCT20191222045857N2). The Institutional Research Ethics Committee, School of Medicine, Tehran University of Medical Sciences, fully approved these investigations (IR.TUMS.SHARIATI.REC.1402.070). All methods were performed following the approved methodology and in accordance with the relevant guidelines and regulations. Each participant provided informed written consent. Furthermore, all participants declared in writing their willingness to participate voluntarily before the start of the study. Informed consent was obtained before any clinical and pictorial data were collected. Consent to publish any personal data was also obtained before manuscript drafting.

## Data Availability

The datasets generated and/or analysed during the current study are available from Hossein Nematian (hosseinnematian76@gmail.com) upon reasonable request.
